# Characterizing female pelvic floor conditions by tactile imaging

**DOI:** 10.1007/s00192-014-2549-9

**Published:** 2014-10-25

**Authors:** Heather van Raalte, Vladimir Egorov

**Affiliations:** 1 Princeton Urogynecology, 601 Ewing Street, Suite B-19, Princeton, NJ 08540 USA; 2 Artann Laboratories, 1459 Lower Ferry Rd., Trenton, NJ 08618 USA

**Keywords:** Biomechanical properties, Vaginal tissue, Tactile imaging, Prolapse, Elasticity

## Abstract

**Introduction and hypothesis:**

Tactile imaging (TI) is the high-definition pressure mapping technology which allows recording pressure patterns from vaginal walls under applied load and during pelvic floor muscle contraction. The objective of this study was to identify new tactile imaging and muscle contraction markers to characterize female pelvic floor conditions.

**Methods:**

The study subjects included 22 women with normal and prolapse conditions. They were examined by a new vaginal tactile imaging probe that images the entire vagina, the pelvic floor support structures, and pelvic floor muscle contractions.

**Results:**

We identified 11 parameters as potential markers to characterize the female pelvic floor conditions. These parameters correlate with prolapse conditions, patient age, and parity.

**Conclusions:**

Our findings suggest that the tactile imaging markers such as pressure, pressure gradient, and dynamic pressure response during muscle contraction may be used for further quantitative characterization of female pelvic floor conditions.

**Electronic supplementary material:**

The online version of this article (doi:10.1007/s00192-014-2549-9) contains supplementary material, which is available to authorized users. This video is also available to watch on http://videos.springer.com/. Please search for the video by the article title.

## Aim of the video

The objective of this study was to identify new tactile imaging and muscle contraction markers to characterize female pelvic floor conditions.

## Methods

Vaginal tactile imaging [1, 2] allows 3-D quantitative elasticity assessment of pelvic floor support structures and carries a potential in assessment of surgical repair. We designed a new vaginal tactile imaging probe that images the entire vagina, the pelvic floor support structures, and pelvic floor muscle contractions. The probe has an orientation sensor, temperature sensors, and 96 pressure sensors positioned every 2.5 mm along both sides of the probe. The examination procedure includes four steps.

Step 1. Probe insertion: This step provides the pressure responses (*P*) for vaginal anterior and posterior compartments along the entire vaginal length. We can use this information to calculate pressure gradients (*grP*) and anatomical dimensions.
Step 2. Probe elevation: This step provides the pressure responses for the apical anterior and posterior compartments that are related to pelvic floor support structures.
Step 3. Probe rotation: This step provides the pressure patterns for the left and right sides of the vagina (circumferential tactile image from vaginal walls).
Step 4. Pelvic floor muscle contractions: This step provides the muscle dynamic pressure responses (*dP*) of the pelvic floor muscle contraction recorded from opposite sides along the entire vaginal length.

In 2013 we enrolled 22 subjects into an observational study (NCT01848626). Two patients were excluded from the data analysis because they had previously had pelvic floor surgery. The analyzed data set included 20 subjects aged from 41 to 70 years. Among them four had normal pelvic floor conditions, four stage I, seven stage II, four stage III, and one stage IV prolapse. A standard physical examination was performed, including a bimanual pelvic examination, Pelvic Organ Prolapse Quantification (POP-Q), assessment of tissue rigidity, and assessment of pelvic floor muscle tone. The tactile imaging data from all examinations were reviewed in a blinded fashion with no knowledge of the subject’s pelvic floor conditions to avoid bias in the data review process. The clinical information was then added to this data set after the tactile imaging data (pressure, pressure gradients, muscle contracting response) were finalized. One-way analysis of variance (ANOVA) (*p*_*a*_), paired *t* test (*p*_*t*_), and Pearson’s correlation coefficients (*r*) were calculated to determine whether the various parameters showed dependence on the pelvic floor conditions. The subjects were asked to complete an assessment of comfort and pain levels for the tactile imaging procedure.

## Results

All 22 patients were successfully examined using the Vaginal Tactile Imager (VTI). We identified the following parameters as potential markers to characterize the female pelvic floor conditions. Site 1 corresponds to the lower one third of the vagina and site 2 to the upper one third of the vagina. We found that nine parameters are sensitive to prolapse conditions (*p* < 0.05 for one-way ANOVA and/or *p* < 0.05 for *t* test with correlation factor *r* from −0.73 to −0.56). These parameters demonstrate a mild-moderate correlation with women age and parity (see Table 1). During step 4 the VTI allows observation of contraction capability of five pelvic floor muscles. Part of the identified markers also demonstrates correlation with patient age and parity. A typical examination consisting of four steps takes 1–2 min. Of the patients, 54 % classified VTI comfort level as more comfortable than manual palpation, 36 % as the same, and 10 % as less comfortable than manual palpation. Of the patients, 73 % classified VTI pain as none, 24 % as mildly painful, and 3 % as a painful.

**Table 1:** Summary for 11 parameters identified as potential markers for pelvic floor characterization

No.	Marker location	Measured/calculated value	Interpretation	Examination step (no.)	One-way ANOVA, *p* vs prolapse staging	*t* test, *p* vs prolapse staging	Correlation coefficient, *r* vs prolapse staging	Correlation coefficient, *r * vs age	Correlation coefficient, *r * vs parity
1	Anterior site 1	Pressure	Tactile feedback	Probe insertion (1)	0.011	0.006	−0.73	−0.17	−0.22
2	Anterior site 1	Pressure gradient	Tissue elasticity	Probe insertion (1)	0.089	0.020	−0.61	−0.13	−0.23
3	Posterior site 1	Pressure	Tactile feedback	Probe insertion (1)	0.008	0.012	−0.71	−0.71–0.39	−0.37
4	Posterior site 1	Pressure gradient	Tissue elasticity	Probe insertion (1)	0.014	0.020	−0.69	−0.40	−0.31
5	Posterior site 1	Pressure	Tactile feedback	Probe elevation (2)	0.030	0.013	0.63	0.52	−0.26
6	Posterior site 2	Pressure	Tactile feedback	Probe rotation (3)	0.35	0.061	−0.40	0.26	0.13
7	Sides site 1	Pressure	Muscle strength	Probe rotation (3)	0.091	0.048	−0.66	−0.26	−0.11
8	Anterior site 1	Pressure change	Muscle strength	Muscle contraction (4)	0.102	0.057	−0.58	0.24	0.00
9	Anterior site 2	Pressure change	Muscle strength	Muscle contraction (4)	0.067	0.016	−0.56	0.11	−0.33
10	Posterior site 1	Pressure change	Muscle strength	Muscle contraction (4)	0.080	0.019	−0.61	0.15	−0.21
11	Posterior site 2	Pressure change	Muscle strength	Muscle contraction (4)	0.046	0.58	0.39	−0.20	−0.27

## Conclusions

Our findings suggest that the tactile imaging markers such as pressure, pressure gradient, and dynamic pressure response during muscle contraction may be used for further quantitative characterization of female pelvic floor conditions.

## Electronic supplementary material

Below is the link to the electronic supplementary material.

ESM 1 (MP4 90696 kb)
